# Radiofrequency Bias Correction of Magnetization Prepared Rapid Gradient Echo MRI at 7.0 Tesla Using an External Reference in a Sequential Protocol

**DOI:** 10.3390/tomography7030038

**Published:** 2021-09-13

**Authors:** Hampus Olsson, Mikael Novén, Jimmy Lätt, Ronnie Wirestam, Gunther Helms

**Affiliations:** 1 Department of Medical Radiation Physics, Clinical Sciences Lund, Lund University, 221 85 Lund, Sweden; ronnie.wirestam@med.lu.se (R.W.); gunther.helms@med.lu.se (G.H.); 2 Department of Linguistics and Phonetics, Lund University, 221 00 Lund, Sweden; mikael.noven@gmail.com; 3 Center for Medical Imaging and Physiology, Skåne University Hospital, 221 00 Lund, Sweden; jimmy.latt@med.lu.se

**Keywords:** bias correction, intensity correction, MPRAGE, MP2RAGE, B1, 7T, ultra-high field, longitudinal relaxation, T1, T1-mapping

## Abstract

At field strengths of 7 T and above, *T*_1_-weighted imaging of human brain suffers increasingly from radiofrequency (RF) *B*_1_ inhomogeneities. The well-known MP2RAGE (magnetization prepared two rapid acquisition gradient echoes) sequence provides a solution but may not be readily available for all MR systems. Here, we describe the implementation and evaluation of a sequential protocol to obtain normalized magnetization prepared rapid gradient echo (MPRAGE) images at 0.7, 0.8, or 0.9-mm isotropic spatial resolution. Optimization focused on the reference gradient-recalled echo (GRE) that was used for normalization of the MPRAGE. A good compromise between white-gray matter contrast and the signal-to-noise ratio (SNR) was reached at a flip angle of 3° and total scan time was reduced by increasing the reference voxel size by a factor of 8 relative to the MPRAGE resolution. The average intra-subject coefficient-of-variation (*CV*) in segmented white matter (WM) was 7.9 ± 3.3% after normalization, compared to 20 ± 8.4% before. The corresponding inter-subject average *CV* in WM was 7.6 ± 7.6% and 13 ± 7.8%. Maps of *T*_1_ derived from forward signal modelling showed no obvious bias after correction by a separately acquired flip angle map. To conclude, a non-interleaved acquisition for normalization of MPRAGE offers a simple alternative to MP2RAGE to obtain semi-quantitative purely *T*_1_-weighted images. These images can be converted to *T*_1_ maps, analogously to the established MP2RAGE approach. Scan time can be reduced by increasing the reference voxel size which has only a miniscule effect on image quality.

## 1. Introduction

The magnetization prepared rapid gradient echo (MPRAGE) sequence has become the standard for structural *T*_1_-weighted (*T*_1_-w) 3D imaging. The *T*_1_ contrast is obtained by an inversion pulse followed by a rapid gradient echo (RAGE) readout with optional delays for free recovery before and after the readout [1]. At ultra-high field (UHF) MRI (7 T or above), the signal-to-noise ratio (SNR) is improved through the increased polarization of nuclear spins, which can be translated into either increased spatial resolution or faster scan times. The former alternative allows for visualization of substructures, unfeasible at lower field strengths [2]. A major challenge of UHF is the increased inhomogeneity of the radiofrequency (RF) *B*_1_ field, which varies based on subject, positioning, and coil [3]. Because of the unpredictability of the *B*_1_ field, pixel values from images acquired at different scanning sessions are generally not reproducible. This means that an important requirement for longitudinal or multi-site studies is not fulfilled. The increased inhomogeneity applies to both the transmit (*B*_1_^+^) and the receive sensitivity components, which combine to form a total bias field, that diminishes the uniformity of the image. To correct for this intensity bias, it was suggested to acquire a reference gradient-recalled echo (GRE) in conjunction with the MPRAGE sequence [4]. Through a simple division of the two acquisitions, a normalized MPRAGE image is obtained where signal variations due to the shared receive coils are eliminated, thus improving image quality as well as reproducibility. *B*_1_^+^ affects the MPRAGE and the GRE pulse sequences differently through the local flip angle and, therefore, the related bias is reduced but not removed in the normalized MPRAGE. The division also removes the influence of proton density (PD) and *T*_2_*, thereby creating a purely *T*_1_-w image with improved tissue contrast. As the pixel values depend on the sequence, the technique is considered to be “semi-quantitative”. The reference GRE can be acquired either separately or interleaved with the MPRAGE at a longer inversion time (TI), and the latter approach has been popularized as the MP2RAGE (magnetization prepared two rapid acquisition gradient echoes) sequence [5].

In this work, we describe an implementation where MPRAGE and reference are acquired in a non-interleaved, sequential fashion to produce the normalized MPRAGE. This approach is an accessible alternative to the interleaved MP2RAGE which, initially, was not readily available on our 7T MR system. Although the non-interleaved variant is expected to be more susceptible to inter-scan subject motion, the reference GRE can be accelerated through enlarged acquisition voxels, allowing for shorter scan time. The optimization procedure focused on the reference GRE, specifically on the flip angle and voxel size, in an effort to improve contrast, SNR, residual *B*_1_^+^ bias, and scan time. The protocol was implemented at three different spatial resolutions, and normalized MPRAGE images acquired at a higher versus a lower spatial resolution were compared. The intra-subject and inter-subject reproducibility (crucial for a semi-quantitative protocol) were also investigated. Finally, the feasibility of calculating *T*_1_ maps from a look-up table (LUT) of the normalized signal, obtained by forward modelling, was explored. The latter approach extends beyond the semi-quantitative domain and results in fully quantitative maps which would allow for a more direct biophysical interpretation in terms of, for instance, myelination [6]. It is analogous to the approach described for the interleaved MP2RAGE by Marques et al. [5]. The work presented here documents an optimized protocol for bias field-corrected structural imaging at 7 T, easily implemented and interpretable by radiologists.

## 2. Materials and Methods

### 2.1. Theory

The influence of receive sensitivity, PD, and *T*_2_* were removed through division of the MPRAGE signal, *S*_MP_, by the reference GRE signal, *S*_GRE_, to yield the normalized MPRAGE signal, *S*_MP/GRE_. This rationale is illustrated by paraphrasing Equation (3) in [4]:

(1) $$S_{\mathrm{MP}/\mathrm{GRE}}=\frac{S_{\mathrm{MP}}}{S_{\mathrm{GRE}}}\propto \frac{f_{R}\rho {\tilde{M}}_{z,\mathrm{MP}}\sin\left(f_{T}\alpha_{\mathrm{MP}}\right)\exp\left(-T_{E}R_{2}^{*}\right)}{f_{R}\rho {\tilde{M}}_{z,\mathrm{GRE}}\sin\left(f_{T}\alpha_{\mathrm{GRE}}\right)\exp\left(-T_{E}R_{2}^{*}\right)}=\frac{{\tilde{M}}_{z,\mathrm{MP}}\sin\left(f_{T}\alpha_{\mathrm{MP}}\right)}{{\tilde{M}}_{z,\mathrm{GRE}}\sin\left(f_{T}\alpha_{\mathrm{GRE}}\right)}$$

where ρ denotes PD, ${\tilde{M}}_{z,\mathrm{MP},\mathrm{GRE}}=M_{z,\mathrm{MP},\mathrm{GRE}}/\rho$ is the longitudinal magnetization per unit PD, $f_{R}$ is a factor accounting for the spatial dependence of receive sensitivity (here, a weighted linear combination of individual channels). The arguments of the sine functions are the local flip angles, i.e., $\alpha_{\mathrm{loc},\mathrm{MP},\mathrm{GE}}=f_{T}\alpha_{\mathrm{MP},\mathrm{GE}}$, where $f_{T}$ takes account of the transmit field (*B*_1_^+^) inhomogeneity. Finally, $R_{2}^{*}=1/T_{2}^{*}$ is the effective transverse relaxation rate. Note that ${\tilde{M}}_{z,\mathrm{MP}}$ is acquired under transient conditions towards a driven equilibrium, ${\tilde{M}}_{0}^{*}$, with an increased rate $R_{1}^{*}=1/T_{1}^{*}$ [7]:

(2) $${\tilde{M}}_{0}^{*}={\tilde{M}}_{0}\cdot \frac{1-\exp\left(-R_{1}TR\right)}{1-\exp\left(-R_{1}^{*}TR\right)}$$

where ${\tilde{M}}_{0}$ is the magnetization at thermal equilibrium $,\;R_{1}^{*}$ is

(3) $$R_{1}^{*}=R_{1}-\ln\left(\cos\left(f_{T}\alpha_{\mathrm{MP}}\right)\right)/TR,$$

and $R_{1}=1/T_{1}$. If full relaxation within one cycle is not obtained, ${\tilde{M}}_{z,\mathrm{MP}}$ will attain a dynamic steady-state between cycles, usually occurring after a few cycles [8]. The degree of agreement between ${\tilde{M}}_{z,\mathrm{MP}}$ and ${\tilde{M}}_{0}^{*}$ when the central k-space line is acquired is a function of TI and $T_{1}$ as well as of the local flip angle and thus $f_{T}$. Note that, without an inversion pulse, ${\tilde{M}}_{z,\mathrm{GRE}}$ is always acquired under steady-state conditions so that

(4) $${\tilde{M}}_{z,\mathrm{GRE}}={\tilde{M}}_{0}^{*}\left(f_{T}\alpha_{\mathrm{GRE}},T_{1}\right).$$

Thus, transmit field related bias may not be removed completely for $\alpha_{\mathrm{MP}}=\alpha_{\mathrm{GRE}}$ as Equation (1) might imply.

### 2.2. Equipment

The protocols were implemented on an actively shielded 7T MR system (Achieva, Philips Healthcare, Best, The Netherlands), using a head coil with two transmitter channels and 32 receive channels (Nova Medical, Wilmington, MA, USA). Healthy subjects were scanned after giving informed written consent and the study was approved by the regional Ethical Review Board. Dielectric pads were used in the experiments [9].

### 2.3. MPRAGE Acquisition

The MPRAGE protocol was built upon the standard protocol for structural MRI available at the research site. Isotropic voxel sizes of either 0.7^3^, 0.8^3^, or 0.9^3^ mm^3^ were acquired with a slab-selective excitation, a readout flip angle of α_MP_ = 8°, TR = 8 ms, fat-water in-phase TE = 1.97 ms and a bandwidth/px of 503 Hz/px. For inversion, an adiabatic pulse with duration 22 ms and a maximal *B*_1_ amplitude of 15 μT was used. The delay from inversion to the central k-space readout (linear phase encoding) was TI = 1200 ms and the time between inversions was T_cycle_ = 3500 ms. These timings (i) allowed for a period of free relaxation after the readout train (increasing dynamic range) and (ii) ensured that the *M*_z_ of cerebrospinal fluid (CSF) was close to the zero-crossing during acquisition of the center of k-space. Both (i) and (ii) will improve *T*_1_ contrast in magnitude images. After each inversion, a single 2D plane of k-space was acquired so that the turbo factor (TF) was identical to the acquisition matrix size in the inner loop phase-encoded direction, *N*_y_. Parallel imaging in the form of sensitivity encoding (SENSE) was applied in the outer loop (right–left direction) with a reduction factor of 2.5 [10]. The inner loop corresponded to the anterior-posterior (AP) direction. Switching between the different resolutions will alter TF and thus affect the *T*_1_ contrast (see Results 4.3). For (0.7 mm)^3^ resolution, a small inner loop SENSE reduction factor of 1.11 had to be applied to fit the readout train within TI = 1200 ms (TF = *N*_y_/1.11). Note that the FOV in the outer loop (FOV_FH,AP,RL_ = 230 × 230 × 180 mm^3^) can be enlarged without affecting contrast. SENSE-related wrap-around artifacts in the AP direction were avoided by an oversampling margin (default setting) which did not affect the acquisition time (*T*_acq_). Lastly, to explore the possibility of further reducing *T*_acq_, the protocol was implemented with elliptical k-space sampling in the phase encoding directions. A prerequisite for this kind of readout is a multi-shot acquisition combined with a zigzag k-space trajectory involving both phase encoding directions during the RAGE readout. This switching between the inner and outer phase encoding loops is performed in such a way that overall contrast is unaffected for constant TF. The acquisition matrix (*N*_x,y,z_), TF and *T*_acq_ for all spatial resolutions with/without elliptical k-space phase encoding are listed in Table 1.

### 2.4. Reference GRE Acquisition

The steady-state GRE sequence was acquired with TR, TE, and outer loop SENSE factor identical to the MPRAGE sequence, but with 50% zero-filling in the outer loop to reduce scan time. In other words, voxel dimensions in the right–left direction were 1.4, 1.6, or 1.8 mm for the (0.7)^3^, (0.8)^3^, and (0.9)^3^ mm^3^ protocols, respectively. When determining the in-plane voxel size (see Results 3.2), the bandwidth/pixel was changed accordingly so that the absolute fat signal displacement was constant between GRE and MPRAGE. Receiver gain and flip angle were calibrated for MPRAGE and then kept constant during the GRE. After acquisition, the volume was reconstructed to the same matrix size as the MPRAGE through zero-filling.

### 2.5. Data Post-processing

Images in the Digital Imaging and Communications in Medicine (DICOM) file format were exported, pseudo-anonymized, and converted to the Neuroimaging Informatics Technology Initiative (NIfTI) file format using an in-house modification of the dcm2niix tool [11]. The platform-dependent scaling of signal intensities was reverted from stored values to 32-bit floating point/1000 (to obtain pixel values in the range of 0–1000) [12]. Spatial dimensions were re-ordered to transverse orientation according to radiological convention (right–left). Rigid co-registration of the reference GRE to the MPRAGE volume was performed using the FMRIB Linear Image Registration Tool (FLIRT) [13,14], where after the normalization was performed as in Equation (1). A mask of the brain was obtained by applying the Brain Extraction Tool (BET) to the PD-w reference GRE [15]. Segmentation of the three major tissue classes, white matter (WM), gray matter (GM) and CSF, was performed using the FMRIB Automated Segmentation Tool (FAST) [16]. Improvement in spatial homogeneity, after signal normalization, for different parameter settings of the reference GRE was analyzed using the coefficient-of-variation (*CV*) over the tissue classes in the normalized MPRAGE. The *CV* within a small WM ROI was used to evaluate relative changes in SNR; the rationale for this approach was that the *CV* in a spatially restricted ROI of homogenous tissue should be unaffected by *B*_1_^+^ and dominated by SNR. Average contrast between WM and GM was defined as $C=\frac{{\bar{S}}_{\mathrm{WM}}-{\bar{S}}_{\mathrm{GM}}}{{\bar{S}}_{\mathrm{WM}}+{\bar{S}}_{\mathrm{GM}}}$ where ${\bar{S}}_{\mathrm{WM}}$ and ${\bar{S}}_{\mathrm{GM}}$ is the average pixel value of the respective segmented tissue type.

### 2.6. Readout Flip Angle of Reference GRE

A higher α_GRE_ will increase the *T*_1_-w of the predominantly PD-w reference GRE, thus reducing tissue contrast in the normalized MPRAGE image. On the other hand, reducing α_GRE_ below the Ernst angle will decrease SNR. To find a compromise between SNR and tissue contrast in the normalized MPRAGE, α_GRE_ was varied from 1° to 6° in increments of 1° in a single subject. In order to obtain comparable pixel values, reference GREs were scaled by a global factor giving a scaled signal

(5) $$S_{\mathrm{GRE},\mathrm{scaled}}\left(\alpha_{\mathrm{GRE},i}\right)=S_{\mathrm{GRE}}\left(\alpha_{\mathrm{GRE},i}\right)/\left(\alpha_{\mathrm{GRE},i}\frac{0.5\cdot \alpha_{\mathrm{GRE},6}^{2}+R_{1}TR}{\alpha_{\mathrm{GRE},6}\left(0.5\alpha_{\mathrm{GRE},i}^{2}+R_{1}TR\right)}\right),$$

where a single $R_{1}=0.83\;s^{-1}$ (corresponding to expected WM $T_{1}\approx 1.2\;s$) was used to approximate the saturation of *M*_z_ [17,18]. The scaling only served to facilitate identical windowing of the images and does not affect the analysis itself. The contrast between segmented WM and GM as well as the *CV* of segmented WM was plotted as a function of α_GRE_. The latter was used as a proxy to evaluate any residual influence of *B*_1_^+^ inhomogeneities.

### 2.7. Voxel Size of Reference GRE

The *B*_1_-related intensity bias (of both receive and transmit effects) comprises mostly low spatial frequencies. Thus, a reference GRE with low spatial resolution is sufficient to correct for *B*_1_ inhomogeneity. On the other hand, this is not the case for PD and *T*_2_* contrast. If the normalized MPRAGE is purposed to produce only semi-quantitative images with a greatly reduced intensity field bias, some dilution of the “pure” *T*_1_ contrast could be acceptable to reduce scan time. In an effort to further reduce scan time and evaluate the effect on the resulting image quality, an MPRAGE volume with 0.7 mm isotropic resolution was normalized by a reference GRE for which the voxel size, *V*_ref_, was varied in-plane as 0.70 × 0.70, 1.05 × 1.05, 1.40 × 1.40, 2.10 × 2.10, and 2.80 × 2.80 mm^2^ (i.e., ×1, ×1.5, ×2, ×3, ×4 the MPRAGE resolution) in a single subject. The voxel dimension in the outer loop (right–left direction) was constant at 1.40 mm resulting in acquisition times of *T*_acq_ = 2:21, 1:35, 1:10, 0:49, and 0:37 min, respectively.

### 2.8. Implementation at Different Resolutions

Two finalized protocols (Results 3.1–3.2) with voxel sizes of (0.7 mm)^3^ and (0.9 mm)^3^ were used on a single subject to compare the effect of the corresponding TF on the contrast. Further, the potential increase of interpolation artifacts at different spatial resolutions (especially in the reference GRE) was of interest to assess.

### 2.9. Intra-Subject Variability

One subject (male, 51 years old, body mass index (BMI) of 23.6) was scanned on five separate occasions over a period of about 8 months using the normalized MPRAGE protocol with 0.7 mm isotropic resolution. Maps of *CV* were compared before and after normalization and the average *CV*s in WM, GM, and CSF were calculated. Regions of interest (ROIs) were manually delineated in the *CV* maps to study local variability. The ROIs were defined in left frontal WM, the left caudate head, the cerebellum, and the left temporal lobe.

### 2.10. Inter-Subject Variability

The 0.8 mm isotropic resolution protocol was previously used in a separate work to study correlations between cortical morphology and language-learning aptitude [19]. From the underlying subject population of this work, a subset of 10 randomly chosen volunteers (7 female, 22 ± 1.9 years old) were used to examine the inter-subject variability of the normalized MPRAGE images. The subjects were all of normal build, that is, height, weight, and BMI were perceived to fall within the 5th and 95th percentiles. Images were diffeomorphically registered to the Montreal Neurological Institute (MNI) space using the diffeomorphic anatomical registration using exponentiated Lie algebra (DARTEL) algorithm as embedded in the statistical parameter mapping (SPM)-based histological MRI (hMRI) toolbox [20,21]. As in the previous subsection, maps of the *CV* were then compared before and after normalization and the average *CV*s in WM, GM, and CSF were calculated. ROIs were likewise defined in MNI space in the same four areas as in the previous subsection, i.e., left frontal WM, left caudate head, cerebellum, and the left temporal lobe. The average *CV*s in WM, GM and CSF were also calculated based on the average pixel intensities of individual segmentations rather than one common segmentation in MNI space.

### 2.11. Comparison to MP2RAGE

The finalized (0.7 mm)^3^ sequential protocol was compared to an interleaved MP2RAGE protocol with the same spatial resolution in one subject [22]. The pulse sequence parameters of the MP2RAGE protocol was as follows: *T*_cycle_/*TI*_1_/*TI*_2_ = 5/0.9/2.75 s, α_1_/α_2_ = 5/3 degrees, TF = 256, TR/TE = 6.8/2.4 ms, bandwidth/px = 365 Hz, *N*_x,y,z_ = 320 × 320 × 256, *T*_acq_ = 8:20 min with a partial Fourier acquisition of 0.75, and a SENSE-factor of 2 in the outer loop right–left direction as well as elliptical k-space phase encoding. The same inversion pulse as in the sequential protocol was used. The MP2RAGE image was calculated using complex data as described in ref. [5] and rigidly co-registered to the sequentially normalized MPRAGE image.

### 2.12. T_1_ Calculation

For proof-of-principle, *T*_1_-mapping using a LUT-based approach was performed on a healthy subject using the (0.8 mm)^3^ resolution protocol together with a DREAM flip angle map [23]. First, the evolution of the longitudinal magnetization, *M*_z_, was simulated for the MPRAGE sequence with imaging parameters as described above, i.e., with α_MP_ = 8°, TR = 8 ms, TI = 1200 ms and TF = 288.

The evolution of *M*_z_ during *T*_cycle_ in the outer loop steady-state (occurring after 2–3 cycles) was simulated using Equations (2) and (3) in the intervals where readout occurred and using normal *T*_1_ relaxation where readout did not occur. The simulations can be performed with ${\tilde{M}}_{z}$ (i.e., per unit ρ). The inversion efficiency applied at the end of each cycle was assumed to be *f*_inv_ = 0.96 [5]. The LUT-derived signal was then calculated for a constant $\alpha_{\mathrm{MP}}$ but over a range of $f_{T}$ as:

(6) $$S_{\mathrm{MP},\mathrm{LUT}}=\frac{{\tilde{M}}_{z,\mathrm{MP}}\left(f_{T},T_{1},TI\right)}{{\tilde{M}}_{0}}\sin\left(f_{T}\alpha_{\mathrm{MP}}\right).$$

The reference GRE was assumed to always be in the inner loop steady-state. Hence, ${\tilde{M}}_{z,\mathrm{GRE}}$ and consequently the LUT-derived GRE signal, $S_{\mathrm{GRE},\mathrm{LUT}}$, is constant for a constant $T_{1}$ and $f_{T}$:

(7) $$S_{\mathrm{GRE},\mathrm{LUT}}=\frac{{\tilde{M}}_{z,\mathrm{GRE}}\left(f_{T},T_{1}\right)}{{\tilde{M}}_{0}}\sin\left(f_{T}\alpha_{\mathrm{MP}}\right).$$

Thus, 2D ($n_{T_{1}}\times n_{f_{T}}$) LUTs of $S_{\mathrm{MP},\mathrm{LUT}}$ and $S_{\mathrm{GRE},\mathrm{LUT}}$ are obtained for a range of $1\;s\leq T_{1}\leq 5\;s$ (step size of 1 ms, $n_{T_{1}}=5000$) and $0.4\leq f_{T}\leq 1.6$ (step size of 0.01, $n_{f_{T}}=121$). The LUTs of the two simulated signals were then combined as follows [5]:

(8) $$S_{\mathrm{MP}2\mathrm{RAGE},\mathrm{LUT}}\left(T_{1},\;f_{T}\right)=\frac{S_{\mathrm{MP},\mathrm{LUT}}\cdot S_{\mathrm{GRE},\mathrm{LUT}}}{S_{\mathrm{MP},\mathrm{LUT}}^{2}+S_{\mathrm{GRE},\mathrm{LUT}}^{2}}.$$

Thus, the values are limited to $0\leq S_{\mathrm{MP}2\mathrm{RAGE},\mathrm{LUT}}\left(T_{1},\;f_{T}\right)\leq 0.5$ in the final LUT for comparison with $S_{\mathrm{MP}2\mathrm{RAGE}}=\frac{S_{\mathrm{MP}}\cdot S_{\mathrm{GRE}}}{S_{\mathrm{MP}}^{2}+S_{\mathrm{GRE}}^{2}}\;$ calculated from the measured magnitude signals. Note that the measured $S_{\mathrm{MP}2\mathrm{RAGE}}\left(T_{1},\;f_{T}\right)$ is limited to positive values for this implementation, since it is not possible to relate the phases of two signals measured sequentially. The $T_{1}$ for which $\left|S_{\mathrm{MP}2\mathrm{RAGE}}-S_{\mathrm{MP}2\mathrm{RAGE},\mathrm{LUT}}\right|$ was minimal was calculated pixelwise either for $f_{T}=1$ or as determined by the DREAM flip angle map.

## 3. Results

### 3.1. Readout Flip Angle of Reference GRE

**Figure 1:**
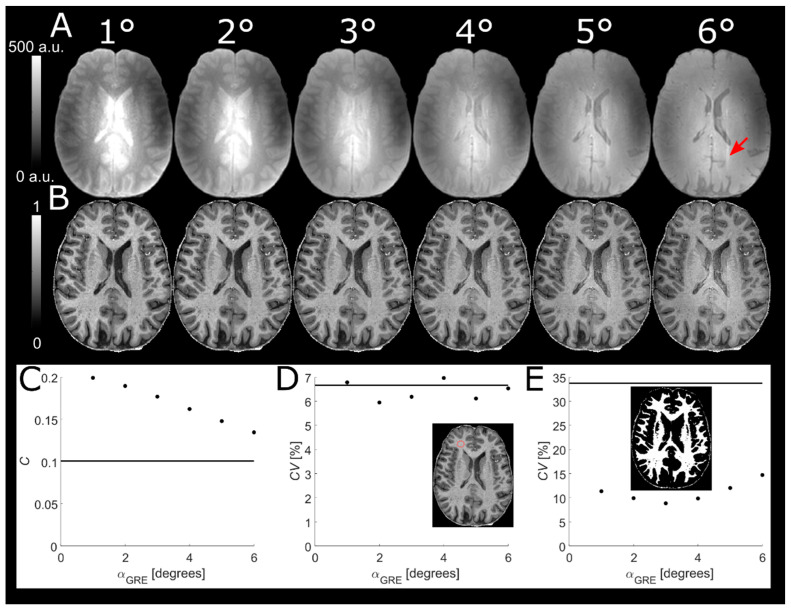
Reference GREs with different α_GRE_ (top row, (**A**)) used to obtain normalized MPRAGE volumes (middle row, (**B**)). Decreasing WM-GM contrast with increasing α_GRE_ is evident from visual inspection of the normalized volumes and verified in the scatter plot (**C**). No change in SNR could be identified by the *CV* in a WM ROI (red circle) (**D**). The *CV* in whole segmented WM had a minimum at α_GRE_ = 3°, implying minimum influence from residual *B*_1_^+^ effects (**E**). These residual *B*_1_^+^ effects are visually identifiable as hyperintense pixels in the center of the normalized MPRAGE for α_GRE_ = 6° (B). The solid lines in the scatter plots represent the respective MPRAGE volume values before normalization. In this experiment, the dielectric pads caused a visible fold-over artefact (red arrow).

There was a continuous decrease in tissue contrast in the normalized volume as α_GRE_ increased (Figure 1). This decrease was evident both from visual inspection (for α_GRE_ > 3°) and from the quantitative comparison of segmented WM and GM (panel C). The contrast was still increased after normalization, compared to before, for all values of α_GRE_. The ROI analysis (panel D) showed no decrease in *CV*, neither at lower α_GRE_ nor after normalization, and no apparent change in SNR could thus be identified. However, a minimum in the *CV* of segmented WM (panel E) was observed at α_GRE_ = 3°, implying minimal influence of residual *B*_1_^+^ inhomogeneities at this setting. The *B*_1_^+^ influence was visually identifiable as elevated pixel values in the center of the brain using α_GRE_ = 6° (panel B). Based on these results, and to avoid deviating too far from the Ernst angle (~7°–5° for 1000 ms ≤ *T*_1_ ≤ 2000 ms), α_GRE_ = 3° was deemed optimal and chosen for the final protocol. At α_GRE_ = 3°, the WM-GM contrast was *C* = 0.18 and the variability across segmented WM was *CV =* 9% after normalization compared to *C* = 0.10 and *CV* = 34% before.

### 3.2. Voxel Size of Reference GRE

**Figure 2:**
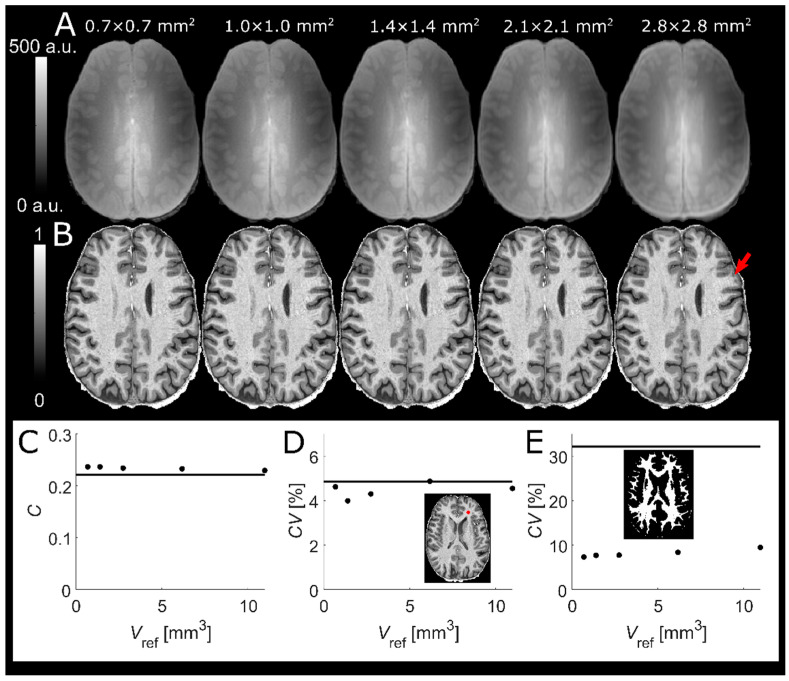
Reference GREs with different in-plane voxel sizes (top row, (**A**))) used to obtain normalized MPRAGE (middle row, (**B**)). The reference GREs and thereby also the normalized MPRAGE volumes are very similar. Some ringing artifacts are visible (red arrow) but do not appear to severely affect image quality even at the lowest resolution in this experiment. Scatter plot of contrast vs. voxel volume (**C**) shows no change in WM-GM contrast with increasing *V*_ref_. No change in SNR could be identified by the *CV* in a WM ROI (red circle) (**D**). A slight increase of the *CV* in the segmented WM is visible at higher *V*_ref_, possibly reflecting PVEs (**E**). The solid lines in the scatter plots represent the respective MPRAGE values before normalization.

Increasing *V*_ref_ (Figure 2) yielded very similar normalized MPRAGE images (panel B). Although some ringing artifacts were visible in the images (cf. red arrow in panel B), increased *V*_ref_ did not cause any noticeably stronger effect. No change in WM-GM contrast (*C* = 0.24 at *V*_ref_ = 0.7 × 0.7 × 1.4 vs. *C* = 0.23 at *V*_ref_ = 2.8 × 2.8 × 1.4) was observed with increasing *V*_ref_ (panel C). Although a higher SNR is to be expected for larger *V*_ref_, no trend in the *CV* of the WM ROI could be discerned (panel D). A weak trend of increasing *CV* in segmented WM was observed (*CV* = 7.3% at *V*_ref_ = 0.7 × 0.7 × 1.4 vs. *CV* = 9.5% at *V*_ref_ = 2.8 × 2.8 × 1.4), possibly reflecting partial volume effects (PVEs) (panel E). No difference between *V*_ref_ = 0.7 × 0.7 × 1.4 and *V*_ref_ = 1.4 × 1.4 × 1.4 was discernible. Hence, *V*_ref_ in one dimension was set to twice that of the MPRAGE voxel size in one dimension, i.e., 1.4^3^/1.6^3^/1.8^3^ mm^3^ for 0.7^3^/0.8^3^/0.9^3^ mm^3^, which reduced the total scan time by 71/53/42 s respectively (i.e., a reduction by 14/14/12%). A higher *V*_ref_ was not employed to avoid more pronounced PVEs and interpolation errors.

### 3.3. Implementation at Different Resolutions

**Figure 3:**
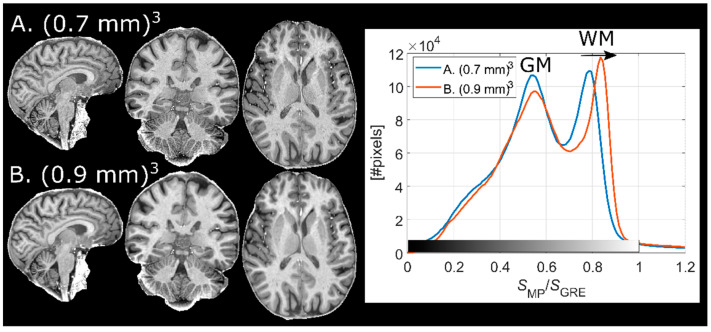
Normalized MPRAGE images acquired on the same subject with two protocols with different resolutions. (**A**) (0.7 mm)^3^, and (**B**) (0.9 mm)^3^. At 0.9 mm isotropic resolution, WM pixel values are increased (black arrow in histogram plot) relative (0.7 mm)^3^ while GM pixel values remain largely unaffected, increasing tissue contrast. This is an effect of the shortened readout train (lower TF).

Figure 3 shows normalized MPRAGE images of the same subject for 0.7 and 0.9-mm isotropic resolution (without elliptical phase encoding). The histograms indicate that signal intensity in GM remains relatively unchanged while WM becomes brighter when the TF is reduced, thus increasing the WM-GM contrast. The reduction in contrast due to higher TF is in concordance with findings by Deichmann et al. [7] No increase in Gibbs ringing at the lower spatial resolution was observed.

### 3.4. Intra-Subject Variability

**Figure 4:**
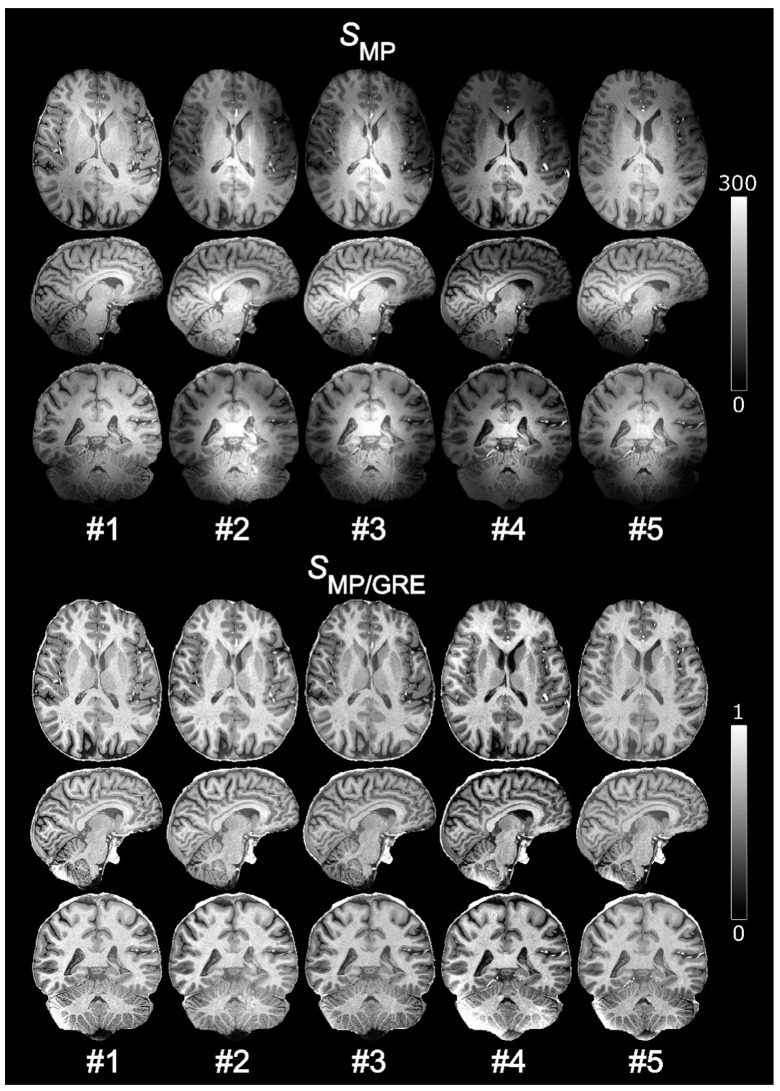
MPRAGE volumes before (*S*_MP_) and after (*S*_MP/GRE_) normalization, acquired on a subject scanned at five separate sessions (day 1, 161, 170, 189, and 253). Influence from the bias field is noticeably reduced after normalization. Hyperintense pixels indicate a failed adiabatic inversion. At the 4th scanning session, there was possibly a transmitter adjustment failure, resulting in generally lower *B*_1_^+^ and thus a generally darker *S*_MP_ image as well as a larger area of very bright pixels in the cerebellumum of *S*_MP/GRE_.

**Figure 5:**
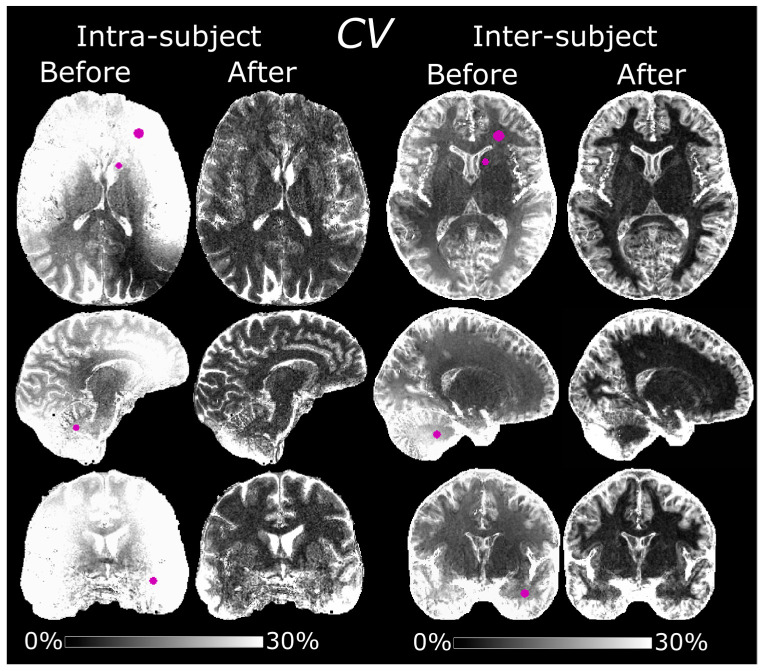
Maps of the *CV* before and after normalization, showing the intra- and inter-subject variability. The intra-subject *CV* maps were calculated from data acquired at five scanning sessions under an eight-month period. The inter-subject *CV* maps, in MNI space, were calculated from data acquired from 10 separate subjects. Reproducibility was improved after normalization in both cases. The ROIs corresponding to the data in Table 2 are denoted in purple in the “Before” maps.

The MPRAGE volumes, before and after normalization, are shown in Figure 4. The normalized volumes show a noticeably increased spatial homogeneity. Hyperintense pixel values in the temporal lobes and cerebellum of the normalized volumes are indicative of a failed inversion with the adiabatic pulse [24]. These bright pixel intensities were exacerbated in session #4, possibly due to a transmitter adjustment failure. Before normalization, the average *CV*s were 20 ± 7.8% in WM, 32 ± 12% in GM and 46 ± 19% in CSF (Figure 5). The corresponding values after normalization were 7.9 ± 3.3% in WM, 15 ± 7.0% in GM and 33 ± 16% in CSF. Exclusion of session #4 yielded an average *CV* of 16 ± 8.1/27 ± 14/39 ± 24% in WM/GM/CSF before normalization and 7.2 ± 2.5/11 ± 6.8/22 ± 14% after. The results of the ROI-based analysis can be seen in Table 2.

### 3.5. Inter-Subject Variability

The *CV* was substantially reduced after normalization also in the inter-subject comparison (Figure 5). This improved reproducibility was most evident in the WM and basal ganglia since the diffeomorphic registration made a pixelwise comparison difficult in the cortex. Before normalization, the average *CV*s in MNI space were 13 ± 7.8/22 ± 9.5/45 ± 20% in WM/GM/CSF, respectively. The corresponding values after normalization were 7.6 ± 7.6/18 ± 10/43 ± 24%. Based on individual segmentations, the *CV*s were 6.2/6.5/9.0% before normalization and 2.0/4.3/6.4% after in WM/GM/CSF. Results of the ROI-based analysis can be seen in Table 2.

### 3.6. Comparison to MP2RAGE

**Figure 6:**
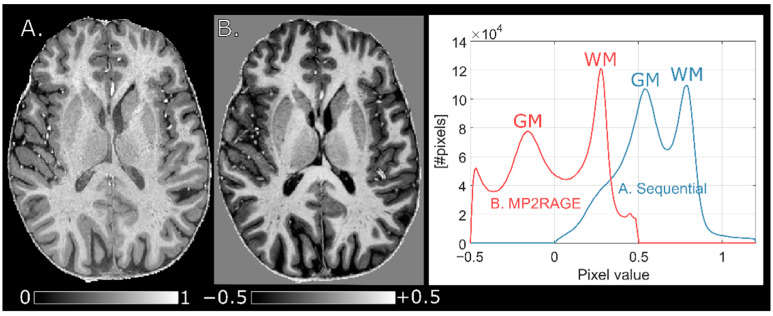
A normalized MPRAGE image (**A**) compared to an MP2RAGE image (**B**) with comparative whole-brain histograms. The MP2RAGE utilizes a larger dynamic range through the interleaved acquisition and complex combination and thus has a higher WM-GM contrast.

The MP2RAGE image showed a higher WM-GM contrast than the normalized MPRAGE obtained using the (0.7 mm)^3^ protocol suggested here (Figure 6). The difference in pixel intensity between the WM/GM histogram modes was 0.785 − 0.545 = 0.24 for the normalized MPRAGE compared to 0.275 − (−0.155) = 0.43 for the MP2RAGE. The underlying reason is the larger dynamic range of *M*_z_ in MP2RAGE. The interleaved acquisition and complex image combination of the MP2RAGE approach can utilize this as information of the polarity of *M*_z_ is retained. The interleaved acquisition of the reference GRE, however, requires a longer *T*_cycle_, which was 5.0 s for MP2RAGE compared to 3.5 s in the sequential protocol. This in turn entails a longer *T*_acq_, which in this case was 8:20 min compared to 5:59 + 1:10 = 7:09 min. The acquisition time of the sequential protocol could have been reduced further to 4:35 + 0:54 = 5:29 min if an elliptical k-space encoding (as was used for the MP2RAGE sequence) had been employed.

### 3.7. T_1_ Calculation

**Figure 7:**
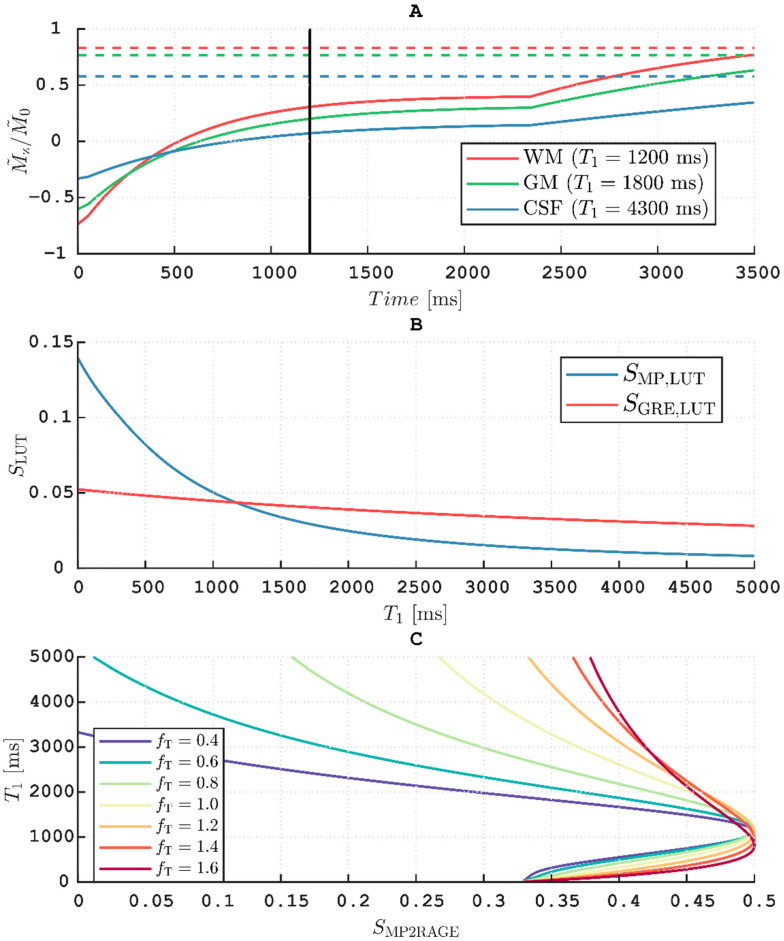
(**A**) Solid coloured lines show the evolution of *M*_z_ during an MPRAGE acquisition across *T*_cycle_ = 3500 ms and *f*_T_ = 1 for three values of *T*_1_ exemplifying WM, GM, and CSF. The dashed lines show the corresponding steady-state of the reference GRE. Vertical black line denotes the center of k-space at TI = 1200 ms. (**B**) The two LUT signals as a function of *T*_1_ for *f*_T_ = 1. (**C**) Estimated *T*_1_ as a function of *S_MP2RAGE_* for different *f*_T_. Areas with longer *T*_1_ were disproportionately biased by deviations in *f*_T_. Depending on *f_T_*, the minimum *T*_1_ that could be uniquely defined ranged from 1181 ms (*f*_T_ = 0.8) and 810 ms (*f*_T_ = 1.6). At *f*_T_ = 0.4, there was also an upper limit at 3330 ms.

**Figure 8:**
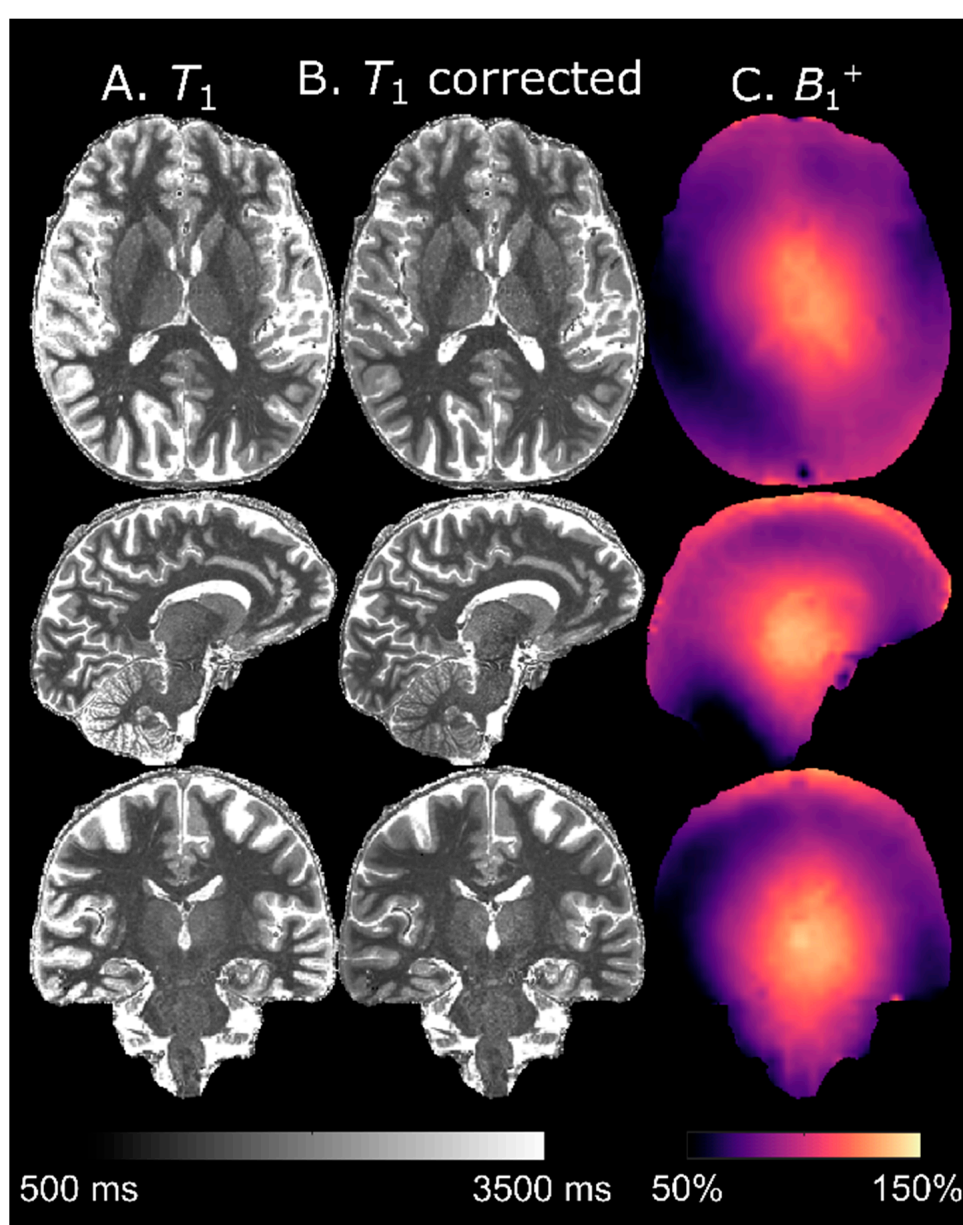
LUT-derived *T*_1_ maps, uncorrected (**A**) and corrected (**B**) with a separately acquired *B*_1_^+^ map (**C**). The *T*_1_ estimation in tissue was moderately robust against *B*_1_^+^ influence and mostly CSF was affected.

The evolution of ${\tilde{M}}_{z,\mathrm{MP}}$ over *T*_cycle_ for TF = 288 is shown in panel A of Figure 7. The LUT signals were derived from this evolution for different *T*_1_s and *f*_T_ = 1 (panel B). The resulting *T*_1_ as a function of *S*_MP2RAGE_ at different *f*_T_ values is also shown (panel C). For these sequence parameters, the *B*_1_^+^ bias was most pronounced at long *T*_1_ (i.e., for CSF). Around approximately 1000 ms, however, *T*_1_ was no longer uniquely defined for a given *S*_MP2RAGE_, which thus set the lower limit of measurable *T*_1_. This lower limit varied, based on *f*_T_ as 1122/1156/1181/1169/1097/966/810 ms for *f*_T_ = 0.4/0.6/0.8/1.0/1.2/1.4/1.6, respectively. At *f*_T_ = 0.4, there was also an upper limit at 3330 ms. Figure 8 shows a map of *T*_1_ derived from this LUT before (panel A) and after (panel B) *B*_1_^+^-correction. Estimated tissue *T*_1_ was only moderately affected by the *B*_1_^+^-correction although an elevation of *T*_1_ in the thalamus (*f*_T_ ≈ 1.3) after correction can be discerned. The most notable difference was seen in the CSF, which was adjusted toward higher values in high *B*_1_^+^ areas and toward lower values in low *B*_1_^+^ areas. Especially after correction, the distribution of *T*_1_ seemed to be very homogenous within different tissues, without any obvious *B*_1_^+^ bias.

### 3.8. Example Results Using the Finalized Protocol

**Figure 9:**
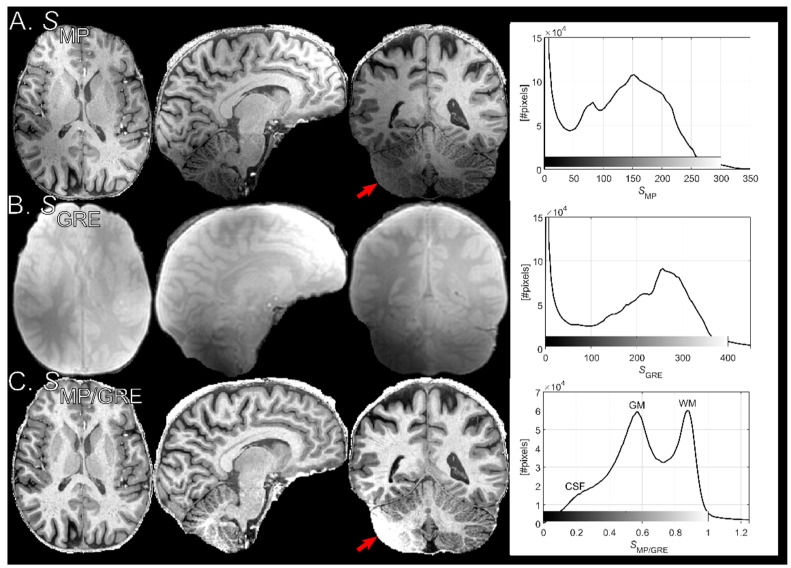
Example images acquired with the MPRAGE protocol before normalization (**A**), the reference GRE used for normalization (**B**), and the MPRAGE image after normalization (**C**). The right-hand panel shows whole-brain histograms of the respective images. Normalization clearly reduced spatial heterogeneity from *B*_1_. The improved homogeneity is also illustrated by the histograms, where GM and WM (and to a lesser extent CSF) modes are visible after normalization. In the right part of the cerebellum (red arrow, coronal plane), *B*_1_^+^ is very weak, leading to failed adiabatic inversion.

Figure 9 shows images from a subject using the finalized protocol (0.8^3^ mm^3^, without elliptical phase encoding). After normalization, effects from the intensity field bias were considerably reduced. For instance, the diagonal *B*_1_^+^ pattern in the axial plane was removed as well as the right–left asymmetry in the coronal plane. The improved homogeneity can also be seen in the whole-brain histogram where WM and GM form distinct modes after normalization. Issues of extremely low *B*_1_^+^ (*f*_T_ ~ 0.3) in the right part of the cerebellum, where the local flip angle is too low to fulfil the adiabatic condition for inversion, can, however, not be resolved by normalization (red arrow).

## 4. Discussion

In this study, we describe the process of implementing a sequential protocol for bias field correction of MPRAGE images at 7 T. Effects of varying the flip angle, different spatial resolutions and acquisition voxel size of the reference GRE were studied, mainly to improve WM-GM contrast and to minimize scan time without introducing biases. The main purpose of the protocols was to obtain semi-quantitative images with “pure” *T*_1_ contrast and improved reproducibility. Improved intra-subject reproducibility was demonstrated by a decreased *CV* of 7.9 ± 3.3% in segmented WM after normalization compared to 20 ± 7.8% before. Likewise, an improved inter-subject reproducibility was demonstrated by the *CV* in segmented WM of 10 subjects which decreased from 13 ± 7.8% to 7.6 ± 7.6%. Local improvement could be higher, for instance 39 ± 3.1% to 7.6 ± 2.2% in frontal WM in the intra-subject experiment and 22 ± 1.4% to 4.4 ± 0.91% in the cerebellum in the inter-subject experiment.

The multiplicative spatial intensity bias imposed by the combination of receive coil signals was removed by normalization. Due to a smaller readout flip angle in the reference GRE compared to the MPRAGE, and to the rather small flip angles overall, the effect of the inhomogeneous transmit field (*B*_1_^+^) was also mostly removed. Since the analytical description of the MPRAGE signal is too complicated to evaluate [25], we estimated *T*_1_ maps through a LUT-based approach analogous to MP2RAGE [5]. As expected, accuracy was improved by using a separate *B*_1_^+^ map.

The protocol was not designed with *T*_1_ calculation, as featured by MP2RAGE, in mind. Thus, at the rather long TI = 1200 ms the protocol fails to effectively exploit the dynamic range obtained when inverting fully relaxed longitudinal magnetization (cf. Figure 7, panel A). This results in quite “saturated” images when normalizing using Equation (8) (pixels close to 0.5), leading to a loss of precision in the *T*_1_ calculation. Further, although whole-brain histograms of *T*_1_ were very similar when using different *V*_ref_, it is most likely prudent to use identical voxel sizes of MPRAGE and the reference GRE if accurate *T*_1_ estimation is of interest, especially at the cortical boundaries that are most susceptible to PVEs. It is important to note that the LUT-based approach assumes that all differences in pixel values are solely caused by variations in *T*_1_ (i.e., that there is “pure” *T*_1_ contrast). By design, the influence of *B*_1_^+^ inhomogeneities on the *T*_1_ calculation is decreased by the normalization of signals. The choice of α_MP_ = 2.7α_GRE_ (8° vs. 3°) appeared to minimize residual effects of *B*_1_^+^ inhomogeneity on the normalized MPRAGE (cf. Figure 1) and thus also on the *T*_1_ calculation. The largest residual effect of *B*_1_^+^ inhomogeneity was found at α_MP_ = 1.3α_GRE_ (8° vs. 6°). This is in concordance with the work of Van de Moortele et al. in which a choice of α_MP_ = α_GRE_ resulted in a much stronger residual *B*_1_^+^ dependence than α_MP_ = 2α_GRE_ [4]. To increase accuracy, a separately acquired flip angle map is still recommended if *T*_1_-mapping is of interest [26]. This is particularly important for longer *T*_1_ values where the *B*_1_^+^ influence is stronger (cf. panel C, Figure 7). The loss of contrast observed in the cerebellum (cf. panel C, Figure 9) occurs when *B*_1_^+^ decreases below the threshold required for the inversion pulse to fulfil the adiabatic condition which cannot be resolved by flip angle mapping [24].

The obvious benefit of an interleaved acquisition is that identical scanning conditions, such as RF power calibration, is guaranteed, as well as increased robustness against inter-scan movement. Inter-scan motion can be corrected by offline rigid coregistration, however. Further, the reduced duration of any one acquisition will reduce the total risk of intra-scan subject movement. The risk of introducing *T*_1_ contrast in the GRE reference due to poor timings (mainly TI and/or TF×TR being too short) is also removed [4]. Importantly, the option to increase *V*_ref_ facilitates the possibility to have a shorter total scan time than needed for the interleaved MP2RAGE.

A protocol with an MPRAGE acquisition voxel size of (0.6 mm)^3^ was also explored (data not shown). However, the SNR in the MPRAGE was deemed unacceptably low. Hence, the (0.7 mm)^3^ protocol here represents the upper limit of the spatial resolution imposed by SNR. It should be noted that noise propagation will moderately decrease the SNR in the normalized MPRAGE, $S_{\mathrm{MP}/\mathrm{GRE}}$, relative to the unnormalized, $S_{\mathrm{MP}}$, by $\frac{S_{\mathrm{MP}}}{\sqrt{1+S_{\mathrm{MP}/\mathrm{GRE}}^{2}}}$, thereby somewhat adversely affecting obtainable spatial resolution [4]. Decreasing the acquisition voxel size also entails increasing TF and thus the duration of the readout train and eventually of TI. This limitation can be circumvented by introducing a SENSE factor in the inner loop, as shown here for (0.7 mm)^3^. The employed zigzag k-space trajectory (see the view-ordering schemes presented for turbo spin echoes in ref. [27]) allows the use of a TF larger than the number of inner loop k-space lines (*N*_y_), which is an effective way to decrease scan time. It further allows the enabling of an elliptical k-space phase encoding which decreases the acquisition time by a factor of approximately $r^{2}/\left(\pi {\left(r/2\right)}^{2}\right)\approx 1.3$ (cf. Table 1). To our knowledge, this kind of k-space trajectory is only readily available on Philips’s systems. Hence, the total scan time of the protocols are supplied with/without elliptical phase encoding.

Correction of RF field-induced bias is recommended at field strengths of 7 T and above. However, normalization may also be necessary to correct for intensity field bias at 3 T, as the wavelength of the *B*_1_ field is approximately 25 cm in brain tissue and thus of the same order of magnitude as the imaged object [28].

## 5. Limitations

We did not attempt to optimize the point-spread function (PSF) of the MPRAGE sequence, although this will be broadened when signal is acquired during a transient state [8]. At increasing resolution, readouts are appended at the beginning and end of the RAGE trains (cf. Figure 7, panel A). These will compromise the PSF, so the effective spatial resolution will not increase as much as the nominal spatial resolution. The increased change in *M*_z_ during acquisition may also influence the WM-GM contrast (cf. Figure 3). In this work, we focused on the reference GRE for normalization, which is acquired entirely in a steady-state and thus does not suffer from such PSF distortions. Further, the PSF cannot be modelled for an elliptical k-space phase encoding, since the trajectory is proprietary.

Like in MP2RAGE, the loss of *T*_1_ contrast at very low *B*_1_^+^ (below the threshold for adiabatic inversion) cannot be mended by normalization [22].

Although subdural ringing artifacts did not noticeably increase when using a protocol with lower spatial resolution (cf. Figure 3), curved ringing artifacts were occasionally observed. These were more evident in the reference GRE but could also be seen in MPRAGE. The artifacts appeared to be correlated to subject movement, but this was not confirmed. These artifacts are believed to be related to interpolation of the low spatial resolution (5.5 × 7.4 × 4.0 mm^3^) SENSE reference scan, acquired prior to the MPRAGE and reference GRE. The artifact was very similar to the “streaky-linear” artifact “type A” described by Sartoretti et al. and showcased in Figure 3, panels (g), (h), and (i) [29].

## 6. Conclusions

We describe a sequential protocol for correction of RF-induced bias in MPRAGE images by normalization with a reference GRE. Spatial homogeneity and WM-GM contrast were improved after normalization as well as intra- and inter-subject reproducibility. Scan time could be reduced by increasing the voxel size of the reference GRE without appreciably affecting image quality.

## Figures and Tables

**Table 1:** Acquisition times (*T*_acq_) of the non-interleaved protocol at different spatial resolutions and with/without elliptical k-space phase encoding. The MPRAGE with (0.7 mm)^3^ resolution used an inner loop SENSE_AP_ = 1.11 to accommodate the readout train.

Parameter/Resolution	(0.7 mm)^3^	(0.8 mm)^3^	(0.9 mm)^3^
MP *N*_x,y,z_	328 × 328 × 257	288 × 288 × 225	256 × 256 × 200
GRE *N*_x,y,z_	164 × 164 × 128	144 × 144 × 112	128 × 128 × 100
MP TF	296	288	256
MP *T*_acq_ (min)	05:59	05:14	04:39
MP *T*_acq_ (elliptical) (min)	04:35	04:04	03:36
GRE *T*_acq_ (min)	01:10	00:53	00:42
GRE *T*_acq_ (elliptical) (min)	00:54	00:40	00:32
Total *T*_acq_ (min)	07:09	06:07	05:21
Total *T*_acq_ (elliptical) (min)	05:45	04:57	04:18

**Table 2:** ROI analysis of the *CV* maps in Figure 5.

ROI	Intra-Subject before	Intra-Subject after Normalization	Inter-Subject before	Inter-Subject after
Frontal WM	39 ± 3.1%	7.6 ± 2.2%	9.2 ± 1.4%	3.7 ± 1.1%
Caudate head	26 ± 2.9%	13 ± 3.5%	6.8 ± 0.90%	3.7 ± 1.2%
Cerebellum	21 ± 3.6%	8.7 ± 2.9%	22 ± 1.4%	4.8 ± 0.91%
Temporal lobe	34 ± 4.3%	9.8 ± 2.3%	16 ± 1.8%	6.1 ± 1.3%

## Data Availability

Data is openly available at https://openneuro.org/datasets/ds003769 (accessed on 10 September 2021). The inter-subject variability data is available as sub-001 to sub-010 at https://openneuro.org/datasets/ds003508 (accessed on 10 September 2021). Descriptive pulse sequence text files as well as a MATLAB scripts to perform the *T*_1_ calculation is available at https://github.com/OlssonHampus/RF_bias_correction_MPRAGE (accessed on 10 September 2021).
